# Genomic Analysis of Indel and SV Reveals Functional and Adaptive Signatures in Hubei Indigenous Cattle Breeds

**DOI:** 10.3390/ani15121755

**Published:** 2025-06-13

**Authors:** Liangyu Shi, Pu Zhang, Bo Yu, Lei Cheng, Sha Liu, Qing Liu, Yuan Zhou, Min Xiang, Pengju Zhao, Hongbo Chen

**Affiliations:** 1 Laboratory of Genetic Breeding, Reproduction and Precision Livestock Farming & Hubei Provincial Center of Technology Innovation for Domestic Animal Breeding, School of Animal Science and Nutritional Engineering, Wuhan Polytechnic University, Wuhan 430023, China; liangyu_shi@whpu.edu.cn (L.S.); z15515092327@163.com (P.Z.); wonderfish@whpu.edu.cn (B.Y.); liusha@163.com (S.L.); liu3996406@163.com (Q.L.); 2 Institute of Animal Science and Veterinary Medicine, Wuhan Academy of Agricultural Sciences, Wuhan 430208, China; chenglei@wuhanagri.com (L.C.); zmax10110@163.com (Y.Z.); xm11609@126.com (M.X.); 3 Hainan Institute, Zhejiang University, Yongyou Industry Park, Yazhou Bay Sci-Tech City, Sanya 572000, China

**Keywords:** Indels, structural variants (SVs), Hubei indigenous cattle, transposable elements, adaptation

## Abstract

The genetic diversity of cattle plays a crucial role in adapting to environmental challenges and enhancing production traits. While research has predominantly focused on single nucleotide polymorphisms (SNPs), small indel and structural variants (SVs) also significantly contribute to genetic variation. This study investigates the distribution and functional impact of insertions and deletions in five Hubei indigenous cattle breeds. A total of 3,208,816 deletions and 2,082,604 insertions were identified, with the majority found in intergenic and intronic regions. Hotspot regions enriched in immune-related genes were identified, underscoring the role of these variants in disease resistance and environmental adaptation. Our analysis revealed a strong influence of transposable elements (TEs), particularly LINEs and SINEs, on genomic rearrangements. The variants were also found to overlap with economically important traits, such as meat quality, reproduction, and immune response. Population structure analysis revealed genetic differentiation among the breeds, with Wuling cattle showing the highest differentiation. Notably, the *NOTCH2* gene was identified as a candidate for regional adaptation due to its significant differentiation across populations. These findings provide valuable genomic resources for enhancing breeding programs, aiming at improving the productivity and resilience of indigenous cattle breeds in China.

## Simple Summary

Understanding genetic variation in cattle is essential for taking advantage of economically important traits such as meat quality, reproduction, and disease resistance. While most studies have focused on single nucleotide polymorphisms (SNPs), this study investigated small indel and structural variants (SVs) across five native cattle breeds from Hubei, China. Whole-genome sequencing of 98 individuals identified over 5 million insertions and deletions, many of which were located in non-coding regions but were still associated with key traits. Several variants, particularly in immune gene-rich regions, were linked to health and meat quality. Our analysis also revealed that transposable elements and simple repeats significantly contributed to these structural differences. A notable insertion in the *NOTCH2* gene, which plays a role in bone remodeling by promoting osteoclast maturation and enhancing their metabolic activity, was validated using PCR. These findings enhance our understanding of structural variation and offer valuable resources for the genetic improvement of Chinese indigenous cattle breeds.

## 1. Introduction

Cattle are essential to rural livelihoods for meat and dairy production, as well as trade worldwide [1,2]. Indigenous cattle breeds are important for genetic resource conservation due to their unique adaptations to local environmental conditions, including disease resistance and environmental adaptation [3,4,5]. Characterizing and conserving these breeds is crucial for understanding their genetic potential and improving livestock production.

Traditionally, genetic research on cattle has focused on single nucleotide polymorphisms (SNPs) to provide insights into the genetic control of traits such as production traits [6,7,8], meat quality [9,10], and disease resistance [11]. Moreover, small insertion-deletions (indels) and structural variants (SVs) also significantly affect phenotypes. Indels and SVs can influence gene dosage, disrupt coding sequences, or modify regulatory regions, thereby affecting gene expression and contributing to various phenotypes [12,13,14]. Moreover, compared to SNPs, indels and SVs affect more base pairs in the genome [15,16]. indels and SVs in immune-related genes, including those in the Jak-STAT and Toll-like receptor pathways, enhance parasite and pathogen resistance [17,18]. Additionally, SVs correlate with ecological gradients such as altitude, temperature, and dry climates, influencing heat tolerance, thermoregulation, and drought resilience [19,20,21]. More importantly, indigenous breeds harbor rare SVs that are mostly absent in commercial breeds, serving as critical reservoirs of adaptive diversity [22,23].

Beyond coding regions, indels and SVs frequently intersect with gene regulatory elements (REs) [24,25,26], thereby modulating gene regulation and splicing. Additionally, transposable elements (TEs), including long interspersed nuclear elements (LINEs) and short interspersed nuclear elements (SINEs), contribute to structural rearrangements by creating novel regulatory sites or disrupting existing ones [27,28]. TEs contribute to insertions and deletions and have shaped the evolution of ruminant interferon (IFN) responses, potentially influencing immune gene regulatory differences across modern breeds [29]. The Bov-tA1 TE has been implicated in immune response and adaptation in global cattle populations [30]. However, explicit analyses of their role in adaptation are limited.

The five Hubei indigenous breeds, situated in the center of China, display comparable production characteristics and overlapping distributions, with minor phenotypic and genetic divergence reported [31]. This study characterized the distribution of indels and SVs across five Hubei indigenous cattle breeds. We identified variation hotspots and explored their functional associations. By annotating the genome, we cataloged indels and SVs, mapped their distribution, and analyzed overlaps with gene structure, QTL, and REs. We further investigated TE-mediated changes and assessed genetic differentiation among these breeds. Our findings reveal genetic differences among Hubei indigenous cattle breeds, which may influence phenotypic traits and local adaptations.

## 2. Materials and Methods

### 2.1. Sample Collection, Genomic Resequencing Read Filtering and Alignment

Ear tissue samples were collected from 80 cattle representing four breeds from Hubei, including Dabieshan (*n* = 28), Wuling (*n* = 14), Yiling (*n* = 20), and Yunba (*n* = 18). The sampled animals were aged between 4 and 60 months. Additionally, sequencing data for the Zaobei breed (*n* = 18) were obtained from a previously published study [32]. All samples were sourced from five core breeding farms in Hubei Province. For each sample, paired-end sequencing libraries were prepared with an average insert size of 500 bp and a read length of 150 bp. High-throughput sequencing was performed using the BGI MGI-T7 platform (MGI Tech Co., Ltd., Shenzhen, China).

Raw sequencing reads underwent quality control using Trimmomatic (v0.39) [33] to remove adapter sequences and low-quality bases, retaining only reads longer than 50 bp with sufficient quality. The filtered reads were then aligned to the *Bos taurus* reference genome (ARS-UCD1.3; GCA_002263795.3) using BWA-MEM (v0.7.17) [34]. The aligned reads were sorted and indexed with Samtools (v1.10) [35], and duplicate reads were marked using GATK MarkDuplicates (v4.1.4.1) [36].

### 2.2. Variant Calling and Filtering

Variant calling analysis includes the detection of single nucleotide polymorphisms (SNPs) and insertions (INSs) and deletions (DELs). The INSs and DELs comprise small indels and structural variations (SVs). All identified indels and SVs were categorized as deletions or insertions and further classified by size: Small (1~10 bp), Medium (11~50 bp), and Large (>50 bp) [37,38].

SNPs and indels calling were performed using HaplotypeCaller [39] in GATK to generate GVCF files for each sample. SNPs and indels were extracted separately using GATK SelectVariants and subjected to quality filtering with GATK VariantFiltration. SNP filtering was based on the following criteria: QualByDepth (QD) < 2.0; Quality (QUAL) < 30.0; StrandOddsRatio (SOR) > 3.0; FisherStrand (FS) > 60.0; RMSMappingQuality (MQ) < 40.0; MappingQualityRankSumTest (MQRankSum) < −12.5; and ReadPosRankSumTest (ReadPosRankSum) < −8.0. Indels were filtered with QD < 2.0, QUAL < 30.0, FS > 200.0, and ReadPosRankSum < −20.0. Only biallelic variants with a missing genotype rate < 0.1 were retained using Bcftools (v1.10.2). Additionally, if an indel was within 10 bp of another indel, the one with the lower QUAL score was removed [40]. These filters were implemented using a custom R script.

**Figure 1:**
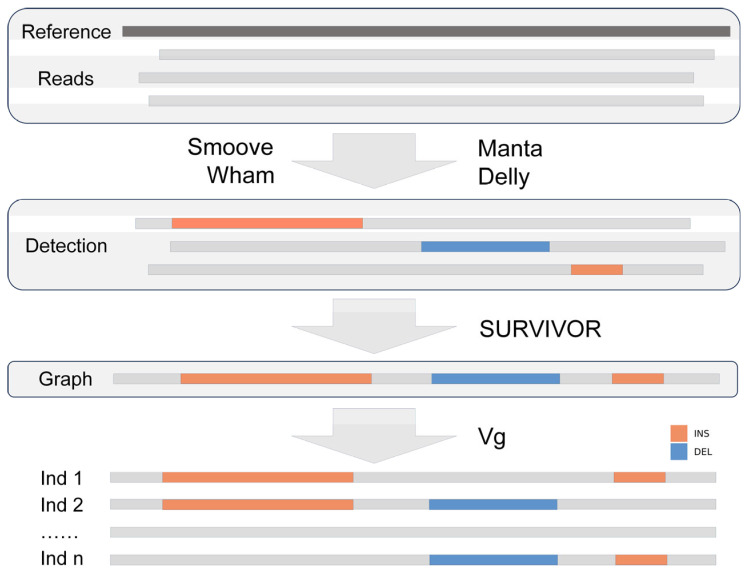
Schematic graph of large deletions (DEL) and insertions (INS).

SVs were detected using a graph-based genotyping strategy (Figure 1). Four software tools were applied with default parameters: Manta (v1.6.0) [41], Delly (v1.3.1) [42], Wham (v1.7.0) [43], and Smoove (v0.2.8) (https://github.com/brentp/smoove/, accessed on 20 April 2025). Only deletions and insertions were identified. SVs of the same type with an overlap greater than 50 bp were merged using SURVIVOR [44]. The candidate SVs were then genotyped with vg software [45,46,47] for each sample, and further filtering was applied to retain only those with a missing rate below 30% and a minor allele frequency (MAF) greater than 0.01 by Vcftools (v0.1.17) [48].

### 2.3. Identification of Insertions and Deletions Hotspots

For insertions and deletions, chromosomes were divided into non-overlapping 100 Kb bins [49]. Regions where the breakpoints ranked in the top 1% were classified as INS and DEL hotspots.

### 2.4. Identification of Genomic Repetitive Sequences in Hubei Cattle

Genomic repetitive sequences, including transposable elements (TE) and tandem repeats, play essential roles in genome evolution and function. Annotation of these sequences was performed using RepeatMasker (v4.1.7) (https://www.repeatmasker.org/, accessed on 20 April 2025) with two reference libraries: RepBase (v201880126) [50] and Dfam (v3.8) [51]. Various TE classes were identified, including DNA transposons, long terminal repeat (LTR) retrotransposons, short interspersed nuclear elements (SINEs), and long interspersed nuclear elements (LINEs). To ensure that genomic repetitive sequences were the primary component of insertions and deletions, only those where the TE length accounted for more than 80% of the SV length were considered in the analysis.

### 2.5. Functional Annotation of Deletions and Insertions in Regulatory and Functional Genomic Regions

Variants annotation was performed using ANNOVAR (v2020Jun08) [44]. Variants were classified into six groups: exonic regions and splice sites, noncoding RNA regions, intronic regions, 5′ and 3′ untranslated regions (UTRs), upstream and downstream regulatory regions, and intergenic regions.

To examine the overlap of indels and SVs with QTLs and regulatory elements (REs), 192,336 QTLs were obtained from the Cattle Quantitative Trait Locus Database (Cattle QTLdb) [52]. The RE dataset [53] included regulatory elements across multiple tissues, such as adipose, cerebellum, cortex, hypothalamus, liver, lung, muscle, and spleen.

To evaluate whether INS and DEL variants overlapped with annotated QTLs in Cattle QTLdb and REs, we performed Z-score calculations and permutation tests using the regioneR package (v1.34.0) [54]. A total of 100 permutations were conducted to assess statistical significance.

### 2.6. Functional Annotation of Indels and SVs in Regulatory and Functional Genomic Regions

We performed linkage disequilibrium (LD) analysis using PLINK to evaluate *r*^2^ between SNPs and indels, as well as SNPs and SVs. Variants were categorized based on *r*^2^: high LD (*r*^2^ ≥ 0.8), medium LD (0.2 ≤ *r*^2^ < 0.8), and low LD (*r*^2^ < 0.2). To further explore regulatory associations, we examined the mapping of SNPs to expression quantitative trait loci (eQTL) and splicing quantitative trait loci (sQTL). eQTL and sQTL data were retrieved from the FarmGTEx database [55], which includes expression data from 37 tissues, such as blood, colon, embryo, kidney, leukocytes, lymph nodes, macrophages, mammary gland, multiple muscle subtypes, reproductive tissues, and various other organs.

### 2.7. Population Structure Analysis

Principal component analysis (PCA) of SNPs, indels, and SVs was carried out using Plink (v1.90) [56]. To assess the genetic relationship between each pair of breeds, pairwise genetic differentiation (*F*_st_) was estimated using Vcftools (v0.1.17) [48]. For different length indel analysis, a sliding window approach was used, with a 50 kb window size and a 20 Kb step. For SV analysis, *F*_st_ base on SV frequencies were calculated within each breed pair. The top 1% of genomic regions were identified as potential selective regions.

### 2.8. Annotation and Enrichment Analysis of Indels and SVs

To investigate the functional enrichment of genes affected by genes located in hotspots and potential selective regions, GO and KEGG pathway analyses were performed using WebGestalt [57,58] (https://www.webgestalt.org/, accessed on 20 April 2025).

### 2.9. PCR Validation of the NOTCH2 67 bp Insertion

To validate the presence of the 67 bp insertion identified in the fourth intron of *NOTCH2*, PCR genotyping was performed using genomic DNA extracted from ear tissue samples of Zaobei, Wuling, and Yunba cattle. A pair of primers flanking the insertion site was designed based on the *Bos taurus* reference genome (ARS-UCD1.3) (forward primer: ACCTTCCAACCAGCAGTGTA; reverse primer: TGGTTGAAGCATGGCCTCTG). The PCR amplification was carried out in a 10 μL reaction system containing 5 μL Taq DNA polymerase (Takara, Shiga, Japan), 3 μL nuclease-free water, 0.5 μL of each primer, and 1 μL of genomic DNA. The cycling conditions included an initial denaturation at 95 °C for 5 min, followed by 35 cycles of 94 °C for 30 s, 62.8 °C for 30 s, and 72 °C for 1 min, with a final extension at 72 °C for 10 min. The PCR products were separated by 2% agarose gel electrophoresis.

## 3. Results

### 3.1. Overview of Resequencing Data and Identified Variants in Hubei Indigenous Cattle

A total of 98 cattle from five indigenous breeds in Hubei Province underwent whole-genome resequencing at an average depth of ~20×, ranging from 17.8× to 28.7×. The mapping rate of reads varied between 97.03% to 99.89%, with an average of 99.72%. The sampled individuals included 25 males and 73 females from five breeds: Dabieshan (*n* = 28), Wuling (*n* = 14), Yiling (*n* = 20), Yunba (*n* = 18), and Zaobei (*n* = 18).

**Figure 2:**
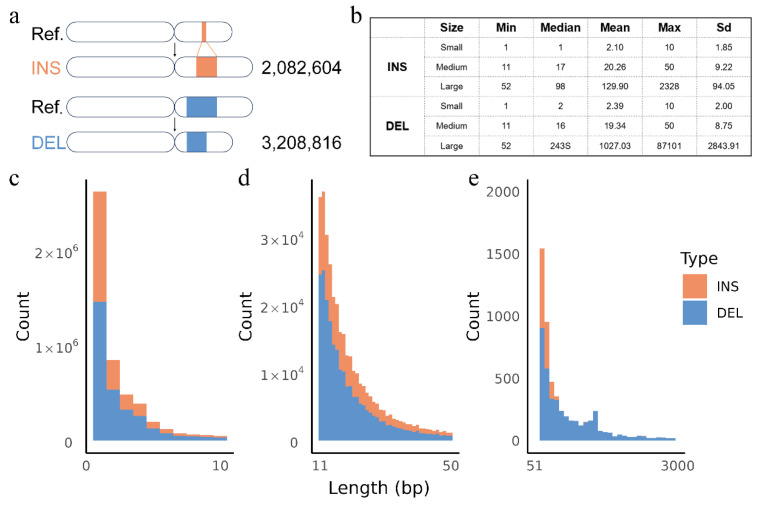
Deletions and insertions distribution in Hubei indigenous cattle: (**a**) the total number of INSs (orange) and DELs (blue); (**b**) the statistics for INSs and DELs; (**c**) stacked histogram of small INSs and DELs (1~10 bp); (**d**) stacked histogram of medium INSs and DELs (11~50 bp); (**e**) stacked histogram of large INSs and DELs (>50 bp).

After quality control, 31,716,252 SNPs, 5,278,767 indels, and 12,653 SVs were identified. To further investigate the distribution patterns of insertions (INSs) and deletions (DELs), 2,082,604 INSs and 3,208,816 DELs were identified (Figure 2a). Small variants accounted for the majority of both INSs and DELs. The average length of small INSs was 2.10 bp, while small DELs averaged 2.39 bp. Large variants exhibited significantly greater lengths and variation, particularly for deletions, which had an average length of 1027.03 bp, with a maximum length of 87,101 bp (Figure 2b). The length distribution of INSs and DELs decreases rapidly with increasing length, with DELs consistently outnumbering INSs across all length categories (Figure 2c–e).

### 3.2. Insertions and Deletions Overlap with Genes, Regulatory Elements and QTLs

**Figure 3:**
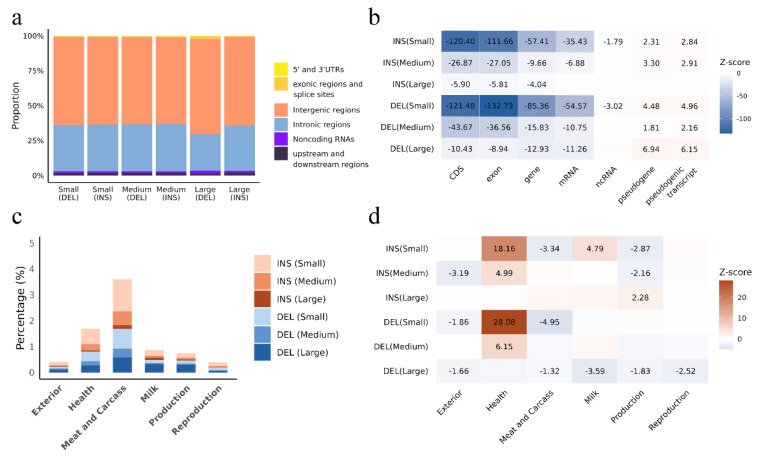
Genomic annotation of insertions and deletions in Hubei indigenous cattle. (**a**) Genomic annotation of INSs and DELs grouped by size. (**b**) Z-score heatmap of INSs and DELs across genomic features. (**c**) Distribution of INSs and DELs overlapping with QTLs related to different trait categories. The Y-axis represents the percentage of INSs and DELs detected for each feature on the X-axis relative to the total number of INSs and DELs. (**d**) Z-score heatmap of INSs and DELs across QTLs.

To assess the genomic distribution of INSs and DELs, all identified variants were annotated by genomic region (Figure 3). In total, 44,844 INSs and 71,197 DELs were detected. Most variants were located in intergenic (67.62~76.12%) and intronic regions (15.69~26.44%), while only a small fraction overlapped with exonic regions (0.60~2.75%), untranslated regions (UTRs) (0.39~0.74%), and upstream/downstream regions (1.58~2.99%) (Figure 3a). INSs and DELs were strongly depleted in coding regions (CDS, exon, gene, and mRNA), with Z-scores ranging from −132.73 to −4.04 (Figure 3b). In contrast, pseudogenes and pseudogenic transcripts showed enrichment (Z-scores: 1.81 to 6.94). Small INSs and DELs displayed the depletion in non-coding RNA (ncRNA) regions, with Z-scores of −1.79 and −3.02, respectively.

Overlap analysis between INSs and DELs and the reported QTLs revealed that most detected variants were located within QTL regions. By length, 1.92% of INSs and 1.69% of DELs overlapped with QTLs associated with meat and carcass traits, particularly smaller insertions. This was followed by overlaps with health-related QTLs (1.69%) and milk production traits (0.86%) (Figure 3c). Both INSs and DELs overlapped with QTLs across all major trait categories at rates significantly higher than expected by chance, with notable enrichment in QTLs related to exterior, health, meat and carcass, milk, production, and reproduction traits (Figure 3d). Health QTLs showed the strongest enrichment signals. All length classes of INSs and DELs had positive Z-scores in health QTLs (ranging from 4.99 to 28.08), with small INSs and DELs showing the highest values, indicating strong enrichment in health-related functional regions. In contrast, all variant types showed depletion in exterior, meat and carcass, and reproduction QTLs. For production traits, small and medium INSs showed depletion (Z = −2.87 and −2.16, respectively), large DELs showed weak depletion (Z = −1.83), while large INSs showed enrichment (Z = 2.28). These findings suggest that INSs and DELs may play regulatory roles in the phenotypic expression of these traits.

A total of 42.12 Kb of INSs and 81.71 Kb of DELs overlapped with candidate REs, including 23.64 Kb within genebody (23.64 Kb/149.77 Mb, 0.02%) and 59.36 Kb TSS (59.36 Kb/133.48 Mb, 0.04%). These INSs and DELs exhibited similarly low frequencies across different tissues (Supplementary Figure S1).

### 3.3. Distribution of Insertions and Deletions Hotspots

**Figure 4:**
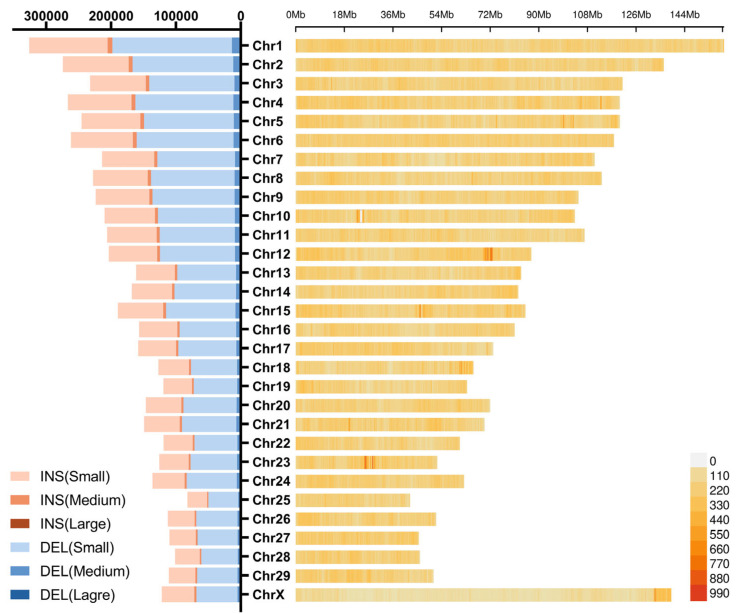
Detection and insertions of indels and SVs in Hubei indigenous cattle breeds.

To characterize the genomic distribution of regions enriched for INSs and DELs, we identified hotspots as genomic regions with a high density of insertions and deletions (Figure 4). A total of 254 hotspots were detected, encompassing 116,040 insertion and deletion variants. The insertions and deletions within these hotspots were most abundant on chromosomes 12, 23, 15, and X, with a clear clustering pattern. By comparing the hotspots with known QTLs, we identified 135 hotspots overlapping with 1594 QTLs, and 76 hotspots for meat and carcass showed the highest hotspot count, including hotspots such as shear force and marbling score.

In the 69.7~72.8 Mb region of chromosome 12, 12,341 insertions and deletions were identified, with annotations for two genes: *TUBGCP3* and *DCUN1D2*. Additionally, ENSBTAG00000026070 was annotated as ncRNA intronic. Two clusters were annotated on chromosome 23, located at 25.6~26.8 Mb and 28.5~30.0 Mb. These regions included two annotated genes: *CARMIL1* and *OR14J1*.

To assess the potential biological implications of these hotspots, we performed GO/KEGG pathway enrichment analyses on genes located within these regions. The analysis of hotspots has a total of 70 GO terms and 26 KEGG pathways (FDR < 0.05) (Table S1).

### 3.4. Repeat-Driven DEL and INSs

**Figure 5:**
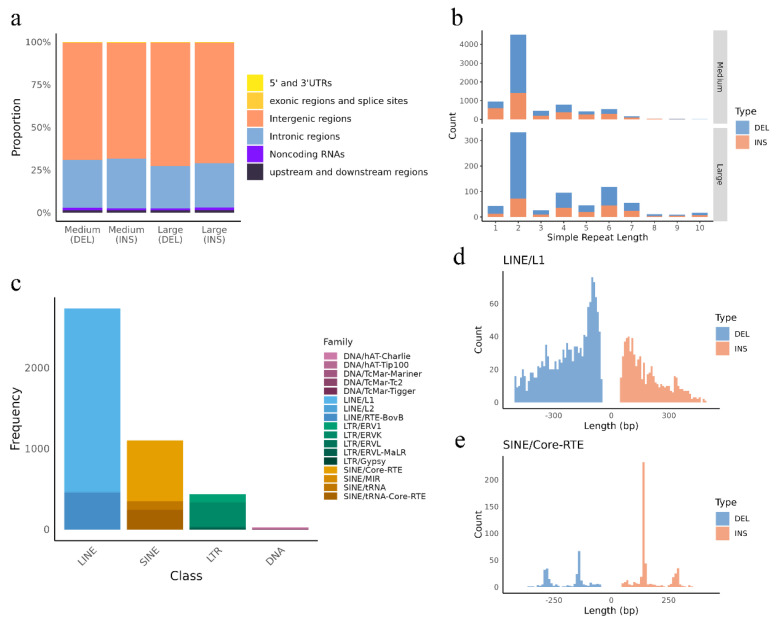
Characterization of TE driving INS and DEL: (**a**) annotation of TEs across genomic regions; (**b**) length distributions of simple repeats; (**c**) frequency distribution of different TE classes; (**d**) length distributions of LINE/L1; (**e**) length distributions of SINE/Core-RTE.

We investigated the role of transposable elements (TEs) and simple repeats in INSs and DELs. These TEs may have influenced gene function by altering regulatory elements, disrupting coding sequences, or facilitating genomic rearrangements (Figure 5). No TEs or simple repeats were detected among small INSs and DELs. A total of 41.79% of the large DELs were driven by TEs, and 45.68% of the large INSs were driven by TEs, mainly located in intergenic (Figure 5a).

A total of 2.20% of the large and medium DELs and 2.92% of the large and medium INSs were associated with simple repeats. Repeat units of length 2 showed the highest frequency of INSs and DELs, with medium DELs being predominant (*n* = 4851). Both INS and DEL counts showed a decreasing trend with increasing repeat unit length from 3 to 10. DELs were consistently more frequent than INSs across all repeat lengths.

LINE and SINE elements were the predominant TE categories, with LINE elements showing the highest frequency. LINE/L1 and SINE/Core-RTE elements were more frequently observed in the 25~50 bp, likely due to the higher abundance of medium-sized INS and DEL in this category. Notably, SINE/Core-RTE elements showed a distinct peak at 150 bp, with most fragments clustering within the 120~150 bp range. Over 98% of these SINE/Core-RTE elements were identified as BOV-A2.

The majority of these TEs and simple repeats were located in intergenic regions. A total of 3194 genes contained these elements. Among them, *PRKG1* had the highest number (23). It was followed by *CSMD3* (20), *PCDH15* (19), and *CTNNA3* (19).

### 3.5. LD-Tag

A total of 9,041,468 SNPs were found to be in LD with INS and DEL related to eQTLs, and 4,700,300 SNPs were in LD linked to sQTLs. Across both eQTL- and sQTL-linked INSs and DELs, small variants (≤10 bp) represented the majority, whereas large variants (>50 bp) were relatively rare. For eQTL-related variants, only five INSs showed low LD with surrounding SNPs. For sQTL-related variants, 1690 INSs and 488 DELs exhibited low LD.

**Figure 6:**
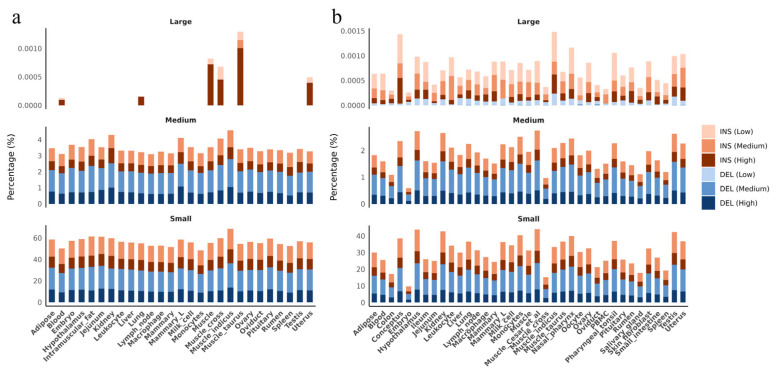
Linkage disequilibrium (LD) patterns of eQTL- and sQTL-associated INS and DEL. (**a**) Tissue-specific distribution of variants linked to eQTLs across different LD categories: high (*r*^2^ ≥ 0.8), medium (0.2 ≤ *r*^2^ < 0.8), and low (*r*^2^ < 0.2). (**b**) Tissue-specific distribution of variants linked to sQTLs. The Y-axis represents the proportion calculated as the total length of adaptive selection regions divided by the total length of each functional category.

Tissue-specific patterns were observed for low-LD variants, particularly in reproductive and metabolic tissues (Figure 6). Among eQTL-linked variants, higher proportions of low-LD variants were found in muscle and mammary tissues, while lower proportions were detected in blood and monocytes (Figure 6a). Large variants contributed only 104 pairs of total LD-tagged variants and were primarily found in muscle and uterus. For sQTL-linked variants, large INSs and DELs showed the highest relative proportion in the low-LD group compared to the medium- and high-LD categories. The highest counts of low-LD large variants were observed in conceptus, muscle, and pharyngeal tonsil.

### 3.6. Population Genetic Differentiation Based on F_st_ Analysis

Principal component analysis (PCA) based on SNPs, indels, and SVs revealed that Dabieshan cattle were the most genetically distinct among the five Hubei indigenous breeds (Supplementary Figure S2). To further investigate population differentiation, pairwise *F*_st_ values were calculated using small, medium, and large INSs and DELs (Supplementary Figures S3–S5). Among the comparisons, the Dabieshan vs. Wuling pair exhibited the highest *F*_st_ values. Overall, the mean pairwise *F*_st_ values indicated low genetic differentiation among the five breeds (Supplementary Figure S6). However, Wuling cattle consistently exhibited slightly higher levels of differentiation from the other breeds. *F*_st_ values for small indels ranged from 0.0040 (Yiling vs. Zaobei) to 0.0323 (Dabieshan vs. Wuling), medium indels from 0.0038 to 0.0296, and large indels from 0.0009 to 0.0208. Across all size ranges, the highest differentiation consistently occurred between Dabieshan and Wuling.

In general, large INSs and DELs showed higher *F*_st_ values compared to medium and small variants. When comparing Wuling cattle to the other breeds, larger variants tended to result in elevated *F*_st_ values. Among all breed comparisons, the Dabieshan vs. Wuling contrast yielded the highest *F*_st_ values across all INSs and DELs classes, indicating substantial genetic divergence between these two populations. Wuling cattle also showed differentiation from Yunba and Yiling breeds, whereas its comparison with Zaobei cattle resulted in relatively lower, but still noticeable, levels of genetic divergence.

To explore potential regions under selection, we identified genes located within the top 1% of *F*_st_ windows for small and medium INSs and DELs, as well as the top 1% of *F*_st_ sites for large INSs and DELs across different size classes (Table 1). Several genes were shared across multiple comparisons. For instance, *UBXN2B* was identified in both the Wuling vs. Yunba and Wuling vs. Yiling comparisons, while *RUNX1* appeared in both the Wuling vs. Dabieshan and Wuling vs. Zaobei comparisons. Notably, the Wuling vs. Zaobei comparison yielded the largest number of shared genes.

### 3.7. NOTCH2 Gene

**Figure 7:**
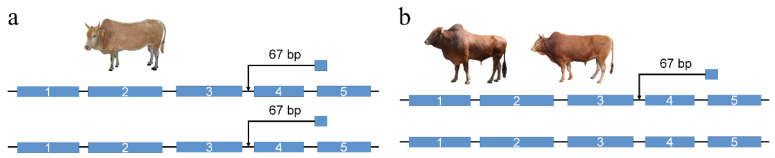
Structural variation of the *NOTCH2* gene in different cattle breeds. (**a**) Zaobei cattle exhibit a homozygous 67 bp insertion within the *NOTCH2* gene. (**b**) Wuling and Yunba cattle display a heterozygous configuration, with the 67 bp insertion. Blue boxes represent exons.

In the *F*_st_ analysis across multiple Hubei indigenous cattle populations, a significant differentiation signal was detected in the *NOTCH2* gene region. A 67 bp INSs located in the fourth intronic regions of *NOTCH2* showed high genetic differentiation between Zaobei and Wuling cattle. Notably, the INS was identified as LINE/L1-derived elements. This gene was also detected in both large-sized *F*_st_ outlier regions when comparing Zaobei cattle with Yunba. The insertion was present on both chromosomes in Zaobei cattle but appeared as a single-copy insertion in Wuling and Yunba (Figure 7). To validate this variant, PCR primers were designed to flank the insertion site, and genotyping was performed across individuals from the three populations (Supplementary Figure S7). These patterns suggest that this insertion represents a population-specific variant in *NOTCH2*, potentially shaped by local adaptation or historical selection pressures.

## 4. Discussion

Structural variants and small indels are increasingly recognized as significant contributors to genetic diversity and phenotypic variation in livestock, such as disease resistance and growth [59]. For example, a 108-bp insertion in *SPN* was linked to tuberculosis resistance in East Asian breeds [20]. Our study provides a detailed characterization of INSs and DELs in five indigenous cattle breeds in Hubei. Indigenous cattle breeds are crucial genetic reservoirs, harboring unique variations associated with adaptation to local environmental stressors such as disease challenges, climatic extremes, and resource limitations. Our analysis offers insights into the importance of these variants in shaping genetic diversity and environmental adaptation. Dabieshan cattle exhibited the highest indel frequency and predominantly deletions. As a representative Chinese indigenous breed, Dabieshan cattle inhabit the surrounding areas of the Dabie Mountains and the middle and lower reaches of the Yangtze River [60]. This elevated mutation numbers might reflect specific adaptive responses to local environmental pressures, given that Dabieshan cattle are widespread across diverse geographical regions including mountainous areas and riverine environments. These unique adaptive pressures likely drive breed-specific evolutionary dynamics.

The distribution of INSs and DELs reflects strong purifying selection, as shown by their depletion in coding regions, likely due to selective pressure against disruptive mutations in essential genes [61,62]. In contrast, their enrichment in pseudogenes and pseudogenic transcripts reflects a possible role in driving pseudogenization [63]. Many pseudogenes originate from INSs and DELs that disrupt gene function [64]. Processed pseudogenes originate from mRNA that lacks regulatory elements, making them nonfunctional from the start [65]. These elements accumulate INSs and DELs faster than functional genes [66], highlighting the role of structural variants in gene inactivation. Similarly, INSs and DELs occurred at low frequencies in regulatory elements (REs), likely due to evolutionary constraints on transcription factor binding site (TFBS) spacing and motif arrangement. Compensatory mechanisms such as enhancer redundancy and TFBS turnover help maintain regulatory function despite sequence variation [67,68,69,70].Trait-specific patterns of enrichment further support the role of INSs and DELs. Health QTLs showed consistent enrichment, especially for small and medium variants, suggesting a potential regulatory role in complex, multifactorial health traits [71]. QTLs associated with reproduction, milk production, and other economically important traits showed depletion, indicating stronger purifying selection in these regions to preserve essential functions [72,73].

The high frequency of INSs and DELs observed in dinucleotide repeats (repeat length = 2) is likely due to replication slippage, a common mechanism in short tandem repeats that promotes strand misalignment during DNA replication [74,75]. In contrast, longer repeat units (3~10 bp) exhibit increased sequence stability and are less prone to such slippage events [76]. Additionally, mismatch repair systems may more effectively recognize and correct errors in longer, more complex repeats [77]. Transposon insertions can disrupt gene function, alter gene expression, and induce chromosomal rearrangements [28]. These effects contribute to genome evolution by introducing genetic variability and structural changes [78]. Genomic hotspot analyses identified chromosomes 12, 23, 15, and X as enriched regions for INSs and DELs, with meat and carcass traits showing the strongest overlap between hotspots and QTLs. In particular, shear force and marbling score accounted for 18 and 14 hotspots, respectively, emphasizing the selective importance of these traits in Hubei beef cattle [79,80].

Variation in body conformation, reproductive performance, and immune regulation in Hubei cattle appear to be interconnected through overlapping genetic pathways. The insertions and deletions identified in this study are concentrated in growth-related genes such as *TUBGCP3* [81,82], *CTNNA3* [83,84,85,86], *CSMD3* [87,88]. A suite of growth- and immune-related genes further modulate reproductive traits. *UBXN2B* overlap QTLs for carcass weight, intramuscular fat deposition and age at first calving, as shown by QTL [89,90] and CNV analyses [91]. Moreover, immune-related genes, including those in the MHC region such as *OR14J1* contribute to immune-reproductive interactions [92]. Functional enrichment analyses point out key pathways, namely, MHC class II complex assembly, peptide antigen binding, and T-cell differentiation, all being critical for embryo implantation, immune tolerance, and pregnancy maintenance. These findings underscore the complex genetic regulation of reproductive traits in cattle. Autoimmune-related pathways, such as systemic lupus erythematosus [93] and type 1 diabetes [94], can disrupt reproductive outcomes by causing immune and endocrine imbalances, potentially increasing the risk of miscarriage and pregnancy complications. The superior immune characteristics of Hubei indigenous cattle are essential for their resilience to local disease challenges. A CNV in *DCUN1D2* is associated with disease resistance [95]. *CARMIL1* plays a role in immune modulation, influencing IL-1-mediated ERK activation [96] and impacting neuroimmune interactions [97].

SVs and small indels that overlap coding exons, promoters, or annotated QTLs represent promising genomic markers for breed identification and selection in indigenous Hubei cattle. This study presents SVs and small indels across five indigenous breeds, providing new insights into genetic diversity. Many polymorphisms are located in loci related to immunity, reproduction, and carcass traits, offering hypotheses for potential trait-associated mechanisms. However, the functional interpretation remains preliminary. Moderate sample sizes per breed limit statistical power. Short-read may fail to detect complex or repetitive structural. In addition, the lack of matched transcriptomic or chromatin-accessibility data limits our ability to infer regulatory impacts in non-coding regions. As a result, many candidate variants are located in intergenic, where their phenotypic effects are likely context-dependent and difficult to detect without integrative data. Future studies should combine long-read sequencing and multi-omics integration. Functional validation approaches such as genome editing will also be essential to confirm causality and identify truly breed-specific loci. Despite current limitations, the dataset presented offers a valuable genomic resource that will support the dissection of adaptive variation and promote precision breeding strategies in Chinese indigenous cattle.

## 5. Conclusions

Genome-wide investigation into insertions and deletions in Hubei indigenous cattle provides insights into adaptation and genetic diversity. We identified 3,208,816 deletions and 2,082,604 insertions across five breeds, revealing hotspots in regions enriched with immune-related genes and pathways. Transposable elements were common and may contribute to local adaptation. Insertions and deletions were associated with traits such as meat quality, disease resistance, and reproduction. Smaller variants were linked to appearance and health, while larger variants were enriched in production-related regions. The *NOTCH2* gene showed high population differentiation and is a potential candidate for adaptation in immune and reproductive pathways. These findings provide valuable genomic resources that can support future breeding strategies to improve livestock productivity and environmental adaptation.

## Figures and Tables

**Table 1:** Shared genes in top 1% *F*_st_ regions across pairwise population comparisons.

Comparison Breed	Shared Genes
Wuling vs. Dabieshan	*RUNX1*, *TRPM3*, *SHISAL2A*
Wuling vs. Yunba	*UBXN2B*, *GLRA3*
Wuling vs. Yiling	*TLN2*, *UBXN2B*
Wuling vs. Zaobei	*AKAP10*, *RUNX1*, *LRRC7*, *LAMA2*, *PIGL*, *PLD1*, *USP25*, *ANO3*, *PLD5*, *MTHFD2L*

## Data Availability

The sequencing data generated in this study have been deposited in the National Genomics Data Center (NGDC) under accession number PRJCA041476.

## Supplementary Materials

The following supporting information can be downloaded at https://www.mdpi.com/article/10.3390/ani15121755/s1, Figure S1. Regulatory element annotation of insertions and deletions in Hubei indigenous cattle; Figure S2. Principal component analysis (PCA) of five indigenous cattle populations based on different variant types; Figure S3. Genome-wide pairwise Fst analysis based on small INSs and DELs among five indigenous cattle breeds; Figure S4. Genome-wide pairwise Fst analysis based on medium INSs and DELs among five indigenous cattle breeds; Figure S5. Genome-wide pairwise Fst analysis based on large INSs and DELs among five indigenous cattle breeds; Figure S6. Mean pairwise Fst values between Hubei indigenous cattle breeds based on different sizes of INSs and DELs; Figure S7. PCR validation of the 67 bp insertion in the NOTCH2 gene across different cattle breeds; Table S1. Summary of GO and KEGG Enrichment Analyses for Genes Within Hotspot Regions.

## References

[1] Gilbert M., Nicolas G., Cinardi G., Van Boeckel T.P., Vanwambeke S.O., Wint G.R.W., Robinson T.P. (2018). Global distribution data for cattle, buffaloes, horses, sheep, goats, pigs, chickens and ducks in 2010. Sci. Data.

[2] Latawiec A.E., Strassburg B.B., Valentim J.F., Ramos F., Alves-Pinto H.N. (2014). Intensification of cattle ranching production systems: Socioeconomic and environmental synergies and risks in Brazil. Animal.

[3] Kim K., Kwon T., Dessie T., Yoo D., Mwai O.A., Jang J., Sung S., Lee S., Salim B., Jung J. (2020). The mosaic genome of indigenous African cattle as a unique genetic resource for African pastoralism. Nat. Genet..

[4] Guan X., Xiang W., Qu K., Ahmed Z., Liu J., Cai M., Zhang J., Chen N., Lei C., Huang B. (2025). Whole genome insights into genetic diversity, introgression, and adaptation of Yunnan indigenous cattle of Southwestern China. BMC Genom..

[5] Buggiotti L., Yurchenko A.A., Yudin N.S., Vander Jagt C.J., Vorobieva N.V., Kusliy M.A., Vasiliev S.K., Rodionov A.N., Boronetskaya O.I., Zinovieva N.A. (2021). Demographic History, Adaptation, and NRAP Convergent Evolution at Amino Acid Residue 100 in the World Northernmost Cattle from Siberia. Mol. Biol. Evol..

[6] Gualdron Duarte J.L., Yuan C., Gori A.S., Moreira G.C.M., Takeda H., Coppieters W., Charlier C., Georges M., Druet T. (2023). Sequenced-based GWAS for linear classification traits in Belgian Blue beef cattle reveals new coding variants in genes regulating body size in mammals. Genet. Sel. Evol..

[7] Niu Q., Zhang T., Xu L., Wang T., Wang Z., Zhu B., Zhang L., Gao H., Song J., Li J. (2021). Integration of selection signatures and multi-trait GWAS reveals polygenic genetic architecture of carcass traits in beef cattle. Genomics.

[8] Sanchez M.P., Tribout T., Kadri N.K., Chitneedi P.K., Maak S., Hoze C., Boussaha M., Croiseau P., Philippe R., Spengeler M. (2023). Sequence-based GWAS meta-analyses for beef production traits. Genet. Sel. Evol..

[9] Fonseca P.A.S., Caldwell T., Mandell I., Wood K., Canovas A. (2022). Genome-wide association study for meat tenderness in beef cattle identifies patterns of the genetic contribution in different post-mortem stages. Meat Sci..

[10] Arikawa L.M., Mota L.F.M., Schmidt P.I., Frezarim G.B., Fonseca L.F.S., Magalhaes A.F.B., Silva D.A., Carvalheiro R., Chardulo L.A.L., Albuquerque L.G. (2024). Genome-wide scans identify biological and metabolic pathways regulating carcass and meat quality traits in beef cattle. Meat Sci..

[11] Twomey A.J., Berry D.P., Evans R.D., Doherty M.L., Graham D.A., Purfield D.C. (2019). Genome-wide association study of endo-parasite phenotypes using imputed whole-genome sequence data in dairy and beef cattle. Genet. Sel. Evol..

[12] Recuerda M., Campagna L. (2024). How structural variants shape avian phenotypes: Lessons from model systems. Mol. Ecol..

[13] Hu D., Zhao Y., Zhu L., Li X., Zhang J., Cui X., Li W., Hao D., Yang Z., Wu F. (2024). Genetic dissection of ten photosynthesis-related traits based on InDel- and SNP-GWAS in soybean. Theor. Appl. Genet..

[14] Luo Y., Zhang M., Guo Z., Wijayanti D., Xu H., Jiang F., Lan X. (2023). Insertion/Deletion (InDel) Variants within the Sheep Fat-Deposition-Related PDGFD Gene Strongly Affect Morphological Traits. Animals.

[15] Das S., Upadhyaya H.D., Srivastava R., Bajaj D., Gowda C.L., Sharma S., Singh S., Tyagi A.K., Parida S.K. (2015). Genome-wide insertion-deletion (InDel) marker discovery and genotyping for genomics-assisted breeding applications in chickpea. DNA Res..

[16] Lecomte L., Arnyasi M., Ferchaud A.L., Kent M., Lien S., Stenlokk K., Sylvestre F., Bernatchez L., Merot C. (2024). Investigating structural variant, indel and single nucleotide polymorphism differentiation between locally adapted Atlantic salmon populations. Evol. Appl..

[17] Vijayakumar P., Singaravadivelan A., Mishra A., Jagadeesan K., Bakyaraj S., Suresh R., Sivakumar T. (2022). Whole-genome comparative analysis reveals genetic mechanisms of disease resistance and heat tolerance of tropical Bos indicus cattle breeds. Genome.

[18] Thambiraja M., Iyengar S.K., Satishkumar B., Kavuru S.R., Katari A., Singh D., Onteru S.K., Yennamalli R.M. (2024). Genetic basis of immunity in Indian cattle as revealed by comparative analysis of *Bos* genome. bioRxiv.

[19] Ben-Jemaa S., Boussaha M., Mandonnet N., Bardou P., Naves M. (2024). Uncovering structural variants in Creole cattle from Guadeloupe and their impact on environmental adaptation through whole genome sequencing. PLoS ONE.

[20] Xia X., Zhang F., Li S., Luo X., Peng L., Dong Z., Pausch H., Leonard A.S., Crysnanto D., Wang S. (2023). Structural variation and introgression from wild populations in East Asian cattle genomes confer adaptation to local environment. Genome Biol..

[21] Ayalew W., Wu X., Tarekegn G.M., Sisay Tessema T., Naboulsi R., Van Damme R., Bongcam-Rudloff E., Edea Z., Enquahone S., Yan P. (2023). Whole-Genome Resequencing Reveals Selection Signatures of Abigar Cattle for Local Adaptation. Animals.

[22] Peripolli E., Stafuzza N.B., Machado M.A., do Carmo Panetto J.C., do Egito A.A., Baldi F., da Silva M. (2023). Assessment of copy number variants in three Brazilian locally adapted cattle breeds using whole-genome re-sequencing data. Anim. Genet..

[23] Pierce M.D., Dzama K., Muchadeyi F.C. (2018). Genetic Diversity of Seven Cattle Breeds Inferred Using Copy Number Variations. Front. Genet..

[24] Wei C., Niu Y., Chen B., Qin P., Wang Y., Hou D., Li T., Li R., Wang C., Yin H. (2022). Genetic effect of an InDel in the promoter region of the NUDT15 and its effect on myoblast proliferation in chickens. BMC Genom..

[25] Wheeler M.M., Stilp A.M., Rao S., Halldorsson B.V., Beyter D., Wen J., Mihkaylova A.V., McHugh C.P., Lane J., Jiang M.Z. (2022). Whole genome sequencing identifies structural variants contributing to hematologic traits in the NHLBI TOPMed program. Nat. Commun..

[26] Wang Y., Shi C., Ge P., Li F., Zhu L., Wang Y., Tao J., Zhang X., Dong H., Gai W. (2023). A 21-bp InDel in the promoter of STP1 selected during tomato improvement accounts for soluble solid content in fruits. Hortic. Res..

[27] Gebrie A. (2023). Transposable elements as essential elements in the control of gene expression. Mob. DNA.

[28] Balachandran P., Walawalkar I.A., Flores J.I., Dayton J.N., Audano P.A., Beck C.R. (2022). Transposable element-mediated rearrangements are prevalent in human genomes. Nat. Commun..

[29] Kelly C.J., Chitko-McKown C.G., Chuong E.B. (2022). Ruminant-specific retrotransposons shape regulatory evolution of bovine immunity. Genome Res..

[30] Zhou Y., Yang L., Han X., Han J., Hu Y., Li F., Xia H., Peng L., Boschiero C., Rosen B.D. (2022). Assembly of a pangenome for global cattle reveals missing sequences and novel structural variations, providing new insights into their diversity and evolutionary history. Genome Res..

[31] Shi L.Y., Zhang P., Yu B., Liu Q., Liu C.H., Lu W., Cheng L., Chen H.B. (2025). Whole-Genome Sequencing Reveals the Role of Cis-Regulatory Elements and eQTL/sQTL in the Adaptive Selection of Hubei Indigenous Cattle. Animals.

[32] Shi L., Zhang P., Liu Q., Liu C., Cheng L., Yu B., Chen H. (2024). Genome-Wide Analysis of Genetic Diversity and Selection Signatures in Zaobei Beef Cattle. Animals.

[33] Bolger A.M., Lohse M., Usadel B. (2014). Trimmomatic: A flexible trimmer for Illumina sequence data. Bioinformatics.

[34] Li H. (2013). Aligning sequence reads, clone sequences and assembly contigs with BWA-MEM. arXiv.

[35] Danecek P., Bonfield J.K., Liddle J., Marshall J., Ohan V., Pollard M.O., Whitwham A., Keane T., McCarthy S.A., Davies R.M. (2021). Twelve years of SAMtools and BCFtools. Gigascience.

[36] Van der Auwera G.A., O’Connor B.D. (2020). Genomics in the Cloud: Using Docker, GATK, and WDL in Terra.

[37] Yang R.D., Nelson A.C., Henzler C., Thyagarajan B., Silverstein K.A.T. (2015). ScanIndel: A hybrid framework for indel detection via gapped alignment, split reads and assembly. Genome Med..

[38] Pokrovac I., Pezer Ä. (2022). Recent advances and current challenges in population genomics of structural variation in animals and plants. Front. Genet..

[39] Poplin R., Ruano-Rubio V., DePristo M.A., Fennell T.J., Carneiro M.O., Van der Auwera G.A., Kling D.E., Gauthier L.D., Levy-Moonshine A., Roazen D. (2017). Scaling accurate genetic variant discovery to tens of thousands of samples. bioRxiv.

[40] Daetwyler H.D., Capitan A., Pausch H., Stothard P., van Binsbergen R., Brondum R.F., Liao X., Djari A., Rodriguez S.C., Grohs C. (2014). Whole-genome sequencing of 234 bulls facilitates mapping of monogenic and complex traits in cattle. Nat. Genet..

[41] Chen X., Schulz-Trieglaff O., Shaw R., Barnes B., Schlesinger F., Kallberg M., Cox A.J., Kruglyak S., Saunders C.T. (2016). Manta: Rapid detection of structural variants and indels for germline and cancer sequencing applications. Bioinformatics.

[42] Rausch T., Zichner T., Schlattl A., Stutz A.M., Benes V., Korbel J.O. (2012). DELLY: Structural variant discovery by integrated paired-end and split-read analysis. Bioinformatics.

[43] Kronenberg Z.N., Osborne E.J., Cone K.R., Kennedy B.J., Domyan E.T., Shapiro M.D., Elde N.C., Yandell M. (2015). Wham: Identifying Structural Variants of Biological Consequence. PLoS Comput. Biol..

[44] Jeffares D.C., Jolly C., Hoti M., Speed D., Shaw L., Rallis C., Balloux F., Dessimoz C., Bahler J., Sedlazeck F.J. (2017). Transient structural variations have strong effects on quantitative traits and reproductive isolation in fission yeast. Nat. Commun..

[45] Garrison E., Sirén J., Novak A.M., Hickey G., Eizenga J.M., Dawson E.T., Jones W., Garg S., Markello C., Lin M.F. (2018). Variation graph toolkit improves read mapping by representing genetic variation in the reference. Nat. Biotechnol..

[46] Sirén J., Monlong J., Chang X., Novak A.M., Eizenga J.M., Markello C., Sibbesen J.A., Hickey G., Chang P.C., Carroll A. (2021). Pangenomics enables genotyping of known structural variants in 5202 diverse genomes. Science.

[47] Hickey G., Heller D., Monlong J., Sibbesen J.A., Sirén J., Eizenga J., Dawson E.T., Garrison E., Novak A.M., Paten B. (2020). Genotyping structural variants in pangenome graphs using the vg toolkit. Genome Biol..

[48] Danecek P., Auton A., Abecasis G., Albers C.A., Banks E., DePristo M.A., Handsaker R.E., Lunter G., Marth G.T., Sherry S.T. (2011). The variant call format and VCFtools. Bioinformatics.

[49] Chen K., Zhang Y., Pan Y., Xiang X., Peng C., He J., Huang G., Wang Z., Zhao P. (2025). Genomic insights into demographic history, structural variation landscape, and complex traits from 514 Hu sheep genomes. J. Genet. Genom..

[50] Bao W., Kojima K.K., Kohany O. (2015). Repbase Update, a database of repetitive elements in eukaryotic genomes. Mob. DNA.

[51] Storer J., Hubley R., Rosen J., Wheeler T.J., Smit A.F. (2021). The Dfam community resource of transposable element families, sequence models, and genome annotations. Mob. DNA.

[52] Hu Z.L., Park C.A., Reecy J.M. (2022). Bringing the Animal QTLdb and CorrDB into the future: Meeting new challenges and providing updated services. Nucleic Acids Res..

[53] Kern C., Wang Y., Xu X., Pan Z., Halstead M., Chanthavixay G., Saelao P., Waters S., Xiang R., Chamberlain A. (2021). Functional annotations of three domestic animal genomes provide vital resources for comparative and agricultural research. Nat. Commun..

[54] Gel B., Díez-Villanueva A., Serra E., Buschbeck M., Peinado M.A., Malinverni R. (2016). regioneR: An R/Bioconductor package for the association analysis of genomic regions based on permutation tests. Bioinformatics.

[55] Liu S., Gao Y., Canela-Xandri O., Wang S., Yu Y., Cai W., Li B., Xiang R., Chamberlain A.J., Pairo-Castineira E. (2022). A multi-tissue atlas of regulatory variants in cattle. Nat. Genet..

[56] Purcell S., Neale B., Todd-Brown K., Thomas L., Ferreira M.A., Bender D., Maller J., Sklar P., De Bakker P.I., Daly M.J. (2007). PLINK: A tool set for whole-genome association and population-based linkage analyses. Am. J. Hum. Genet..

[57] Zhang B., Kirov S., Snoddy J. (2005). WebGestalt: An integrated system for exploring gene sets in various biological contexts. Nucleic Acids Res..

[58] Elizarraras J.M., Liao Y., Shi Z., Zhu Q., Pico A.R., Zhang B. (2024). WebGestalt 2024: Faster gene set analysis and new support for metabolomics and multi-omics. Nucleic Acids Res..

[59] Talenti A., Powell J., Wragg D., Chepkwony M., Fisch A., Ferreira B.R., Mercadante M.E.Z., Santos I.M., Ezeasor C.K., Obishakin E.T. (2022). Optical mapping compendium of structural variants across global cattle breeds. Sci. Data.

[60] Guan X.W., Zhao S.P., Xiang W.X., Jin H., Chen N.B., Lei C.Z., Jia Y.T., Xu L. (2022). Genetic Diversity and Selective Signature in Dabieshan Cattle Revealed by Whole-Genome Resequencing. Biology.

[61] de la Chaux N., Messer P.W., Arndt P.F. (2007). DNA indels in coding regions reveal selective constraints on protein evolution in the human lineage. Bmc Evol. Biol..

[62] Yang Y., Braga M., Dean M.D. (2024). Insertion-Deletion Events Are Depleted in Protein Regions with Predicted Secondary Structure. Genome Biol. Evol..

[63] Qian S.H., Chen L., Xiong Y.L., Chen Z.X. (2022). Evolution and function of developmentally dynamic pseudogenes in mammals. Genome Biol..

[64] Tutar Y. (2012). Pseudogenes. Comp. Funct. Genom..

[65] Esnault C., Maestre J., Heidmann T. (2000). Human LINE retrotransposons generate processed pseudogenes. Nat. Genet..

[66] Zhang Z., Carriero N., Gerstein M. (2004). Comparative analysis of processed pseudogenes in the mouse and human genomes. Trends Genet..

[67] Cameron R.A., Chow S.H., Berney K., Chiu T.Y., Yuan Q.A., Kramer A., Helguero A., Ransick A., Yun M., Davidson E.H. (2005). An evolutionary constraint: Strongly disfavored class of change in DNA sequence during divergence of cis-regulatory modules. Proc. Natl. Acad. Sci. USA.

[68] Martinez C., Rest J.S., Kim A.R., Ludwig M., Kreitman M., White K., Reinitz J. (2014). Ancestral resurrection of the Drosophila S2E enhancer reveals accessible evolutionary paths through compensatory change. Mol. Biol. Evol..

[69] Barriere A., Gordon K.L., Ruvinsky I. (2012). Coevolution within and between regulatory loci can preserve promoter function despite evolutionary rate acceleration. PLoS Genet..

[70] Kliesmete Z., Orchard P., Lee V.Y.K., Geuder J., Krauss S.M., Ohnuki M., Jocher J., Vieth B., Enard W., Hellmann I. (2024). Evidence for compensatory evolution within pleiotropic regulatory elements. Genome Res..

[71] Chiang C., Scott A.J., Davis J.R., Tsang E.K., Li X., Kim Y., Hadzic T., Damani F.N., Ganel L., Montgomery S.B. (2017). The impact of structural variation on human gene expression. Nat. Genet..

[72] Sudmant P.H., Rausch T., Gardner E.J., Handsaker R.E., Abyzov A., Huddleston J., Zhang Y., Ye K., Jun G., Fritz M.H.Y. (2015). An integrated map of structural variation in 2504 human genomes. Nature.

[73] Ruderfer D.M., Hamamsy T., Lek M., Karczewski K.J., Kavanagh D., Samocha K.E., Daly M.J., MacArthur D.G., Fromer M., Purcell S.M. (2016). Patterns of genic intolerance of rare copy number variation in 59,898 human exomes. Nat. Genet..

[74] Levinson G., Gutman G.A. (1987). Slipped-strand mispairing: A major mechanism for DNA sequence evolution. Mol. Biol. Evol..

[75] Ellegren H. (2004). Microsatellites: Simple sequences with complex evolution. Nat. Rev. Genet..

[76] Gemayel R., Cho J., Boeynaems S., Verstrepen K.J. (2012). Beyond Junk-Variable Tandem Repeats as Facilitators of Rapid Evolution of Regulatory and Coding Sequences. Genes.

[77] Miller C.J., Usdin K. (2022). Mismatch repair is a double-edged sword in the battle against microsatellite instability. Expert. Rev. Mol. Med..

[78] Lawson H.A., Liang Y.H., Wang T. (2023). Transposable elements in mammalian chromatin organization. Nat. Rev. Genet..

[79] Qiu X., Qin X., Chen L., Chen Z., Hao R., Zhang S., Yang S., Wang L., Cui Y., Li Y. (2022). Serum Biochemical Parameters, Rumen Fermentation, and Rumen Bacterial Communities Are Partly Driven by the Breed and Sex of Cattle When Fed High-Grain Diet. Microorganisms.

[80] Wei M., Liu X., Xie P., Lei Y., Yu H., Han A., Xie L., Jia H., Lin S., Bai Y. (2022). Characterization of Volatile Profiles and Correlated Contributing Compounds in Pan-Fried Steaks from Different Chinese Yellow Cattle Breeds through GC-Q-Orbitrap, E-Nose, and Sensory Evaluation. Molecules.

[81] Luo C., Xu X., Zhao C., Wang Q., Wang R., Lang D., Zhang J., Hu W., Mu Y. (2024). Insight Into Body Size Evolution in Aves: Based on Some Body Size-Related Genes. Integr. Zool..

[82] Deng M.T., Zhu F., Yang Y.Z., Yang F.X., Hao J.P., Chen S.R., Hou Z.C. (2019). Genome-wide association study reveals novel loci associated with body size and carcass yields in Pekin ducks. BMC Genom..

[83] Janssens B., Mohapatra B., Vatta M., Goossens S., Vanpoucke G., Kools P., Montoye T., van Hengel J., Bowles N.E., van Roy F. (2003). Assessment of the CTNNA3 gene encoding human alpha T-catenin regarding its involvement in dilated cardiomyopathy. Hum. Genet..

[84] Zhao L., Li F., Yuan L., Zhang X., Zhang D., Li X., Zhang Y., Zhao Y., Song Q., Wang J. (2022). Expression of ovine CTNNA3 and CAP2 genes and their association with growth traits. Gene.

[85] Sun X., Niu Q., Jiang J., Wang G., Zhou P., Li J., Chen C., Liu L., Xu L., Ren H. (2023). Identifying Candidate Genes for Litter Size and Three Morphological Traits in Youzhou Dark Goats Based on Genome-Wide SNP Markers. Genes.

[86] Yu H.W., Yu S.C., Guo J.T., Cheng G., Mei C.G., Zan L.S. (2023). Genome-Wide Association Study Reveals Novel Loci Associated with Body Conformation Traits in Qinchuan Cattle. Animals.

[87] Del Gobbo G.F., Yin Y., Choufani S., Butcher E.A., Wei J., Rajcan-Separovic E., Bos H., von Dadelszen P., Weksberg R., Robinson W.P. (2021). Genomic imbalances in the placenta are associated with poor fetal growth. Mol. Med..

[88] Teodoro M., Maiorano A.M., Campos G.S., de Albuquerque L.G., de Oliveira H.N. (2025). Genetic parameters, genomic prediction, and identification of regulatory regions located on chromosome 14 for weight traits in Nellore cattle. J. Anim. Breed. Genet..

[89] Alam M.Z., Haque M.A., Iqbal A., Lee Y.M., Ha J.J., Jin S., Park B., Kim N.Y., Won J.I., Kim J.J. (2023). Genome-Wide Association Study to Identify QTL for Carcass Traits in Korean Hanwoo Cattle. Animals.

[90] Haque M.A., Lee Y.M., Ha J.J., Jin S., Park B., Kim N.Y., Won J.I., Kim J.J. (2024). Genome-wide association study identifies genomic regions associated with key reproductive traits in Korean Hanwoo cows. BMC Genom..

[91] Wang Y., Ma J., Wang J., Zhang L., Xu L., Chen Y., Zhu B., Wang Z., Gao H., Li J. (2024). Genome-Wide Detection of Copy Number Variations and Their Potential Association with Carcass and Meat Quality Traits in Pingliang Red Cattle. Int. J. Mol. Sci..

[92] Jahromi M.M. (2012). Haplotype specific alteration of diabetes MHC risk by olfactory receptor gene polymorphism. Autoimmun. Rev..

[93] Tan Y., Yang S., Liu Q., Li Z., Mu R., Qiao J., Cui L. (2022). Pregnancy-related complications in systemic lupus erythematosus. J. Autoimmun..

[94] Taylor R., Davison J.M. (2007). Type 1 diabetes and pregnancy. BMJ.

[95] Wu Q.d., Zhou Y.d., Wang Y., Zhang Y., Shen Y., Su Q., Gao G., Xu H., Zhou X., Liu B. (2020). Whole-genome sequencing reveals breed-differential CNVs between Tongcheng and Large White pigs. Anim. Genet..

[96] Wang Q., Notay K., Downey G.P., McCulloch C.A. (2020). The Leucine-Rich Repeat Region of CARMIL1 Regulates IL-1-Mediated ERK Activation, MMP Expression, and Collagen Degradation. Cell Rep..

[97] Chen Q., Qu K., Ma Z., Zhan J., Zhang F., Shen J., Ning Q., Jia P., Zhang J., Chen N. (2020). Genome-Wide Association Study Identifies Genomic Loci Associated With Neurotransmitter Concentration in Cattle. Front. Genet..

