# Supplementary material for: Genomic Analysis of Indel and SV Reveals Functional and Adaptive Signatures in Hubei Indigenous Cattle Breeds
Source: Animals (Basel). 2025 Jun 13;15(12):1755. doi: 10.3390/ani15121755 (PMC12189102; doi:10.3390/ani15121755)
Supplement: Supplementary file 1 [file animals-15-01755-s001.zip › Figures S1-S7.pdf]

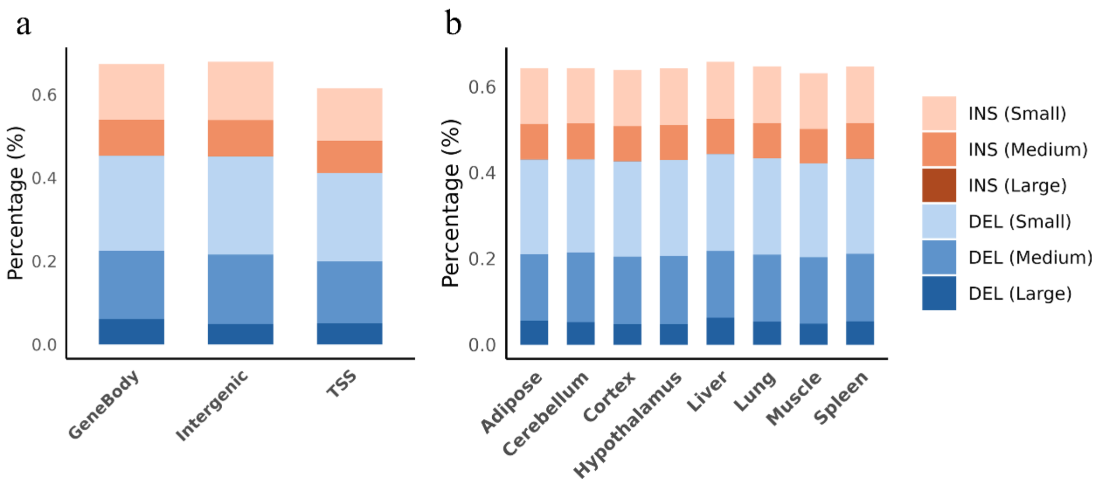

Supplementary Figure S1. Regulatory element annotation of insertions and deletions in Hubei indigenous cattle. (**a**) Proportion of INSs and DELs overlapping with REs. The Y-axis represents the proportion calculated as the total length of INSs and DELs divided by the total length of each functional category. (**b**) Distribution of INSs and DELs overlapping with REs across different tissues. The Y-axis represents the proportion calculated as the total length of INSs and DELs divided by the total length of each functional category.

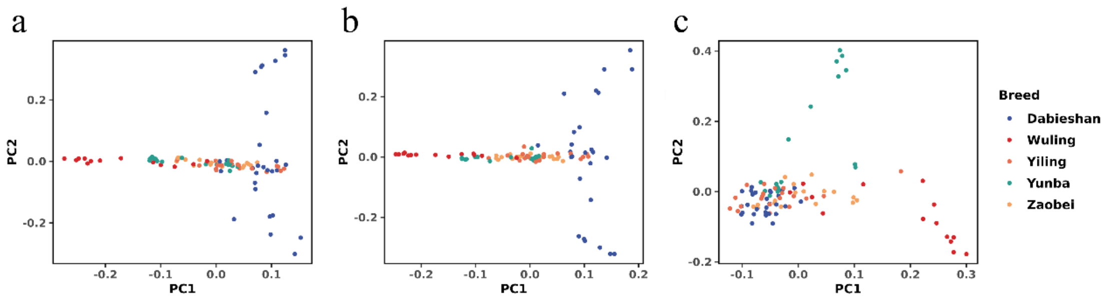

Supplementary Figure S2. Principal component analysis (PCA) of five indigenous cattle populations based on different variant types: (**a**) PCA based on SNPs; (**b**) PCA based on indels; (**c**) PCA based on SVs.

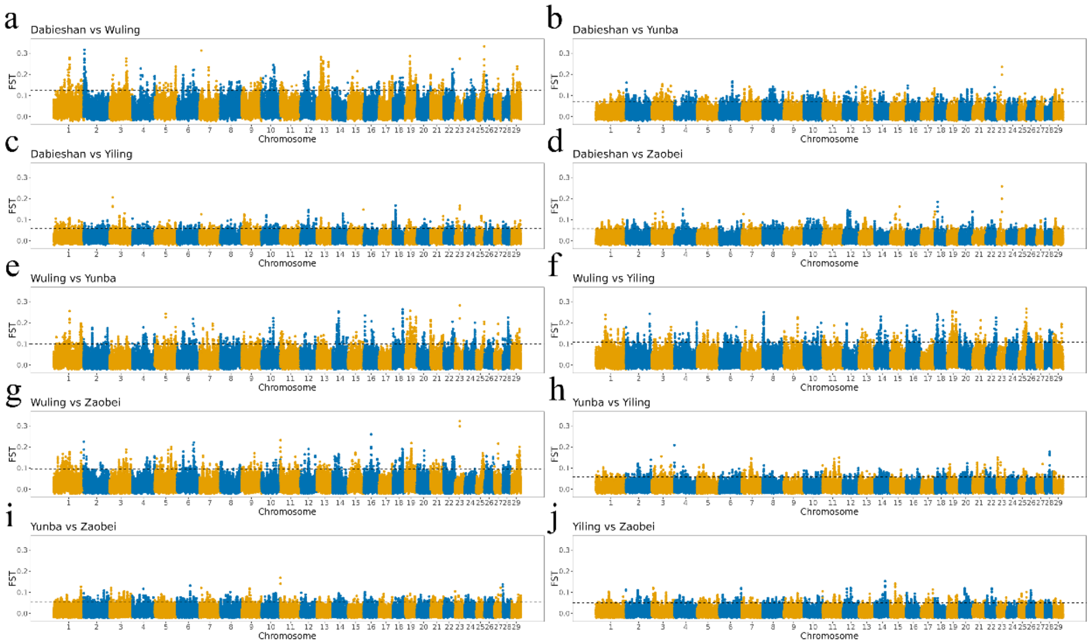

Supplementary Figure S3. Genome-wide pairwise F_{st} analysis based on small INSs and DELs among five indigenous cattle breeds: (**a**) Dabieshan vs. Wuling; (**b**) Dabieshan vs. Yunba; (**c**) Dabieshan vs. Yiling. (**d**) Dabieshan vs. Zaobei; (**e**) Wuling vs. Yunba; (**f**) Wuling vs. Yiling; (**g**) Wuling vs. Zaobei; (**h**) Yunba vs. Yiling. (**i**) Yunba vs. Zaobei; (**j**) Yiling vs. Zaobei.

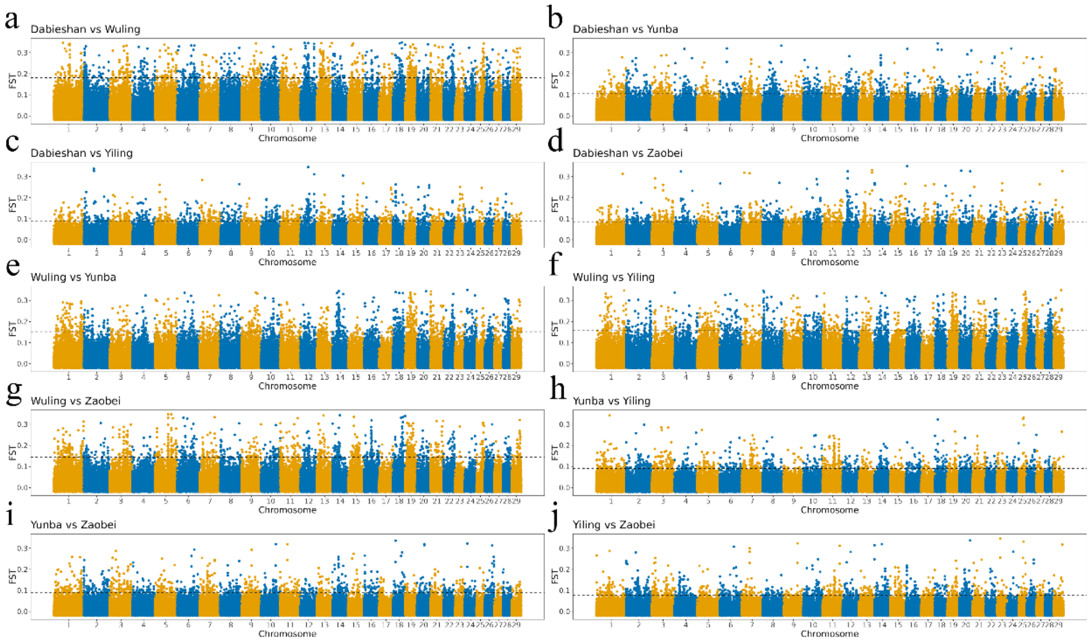

Supplementary Figure S4. Genome-wide pairwise F_{st} analysis based on medium INSs and DELs among five indigenous cattle breeds: (**a**) Dabieshan vs. Wuling. (**b**) Dabieshan vs. Yunba; (**c**) Dabieshan vs. Yiling; (**d**) Dabieshan vs. Zaobei; (**e**) Wuling vs. Yunba; (**f**) Wuling vs. Yiling; (**g**) Wuling vs. Zaobei; (**h**) Yunba vs. Yiling; (**i**) Yunba vs. Zaobei; (**j**) Yiling vs. Zaobei.

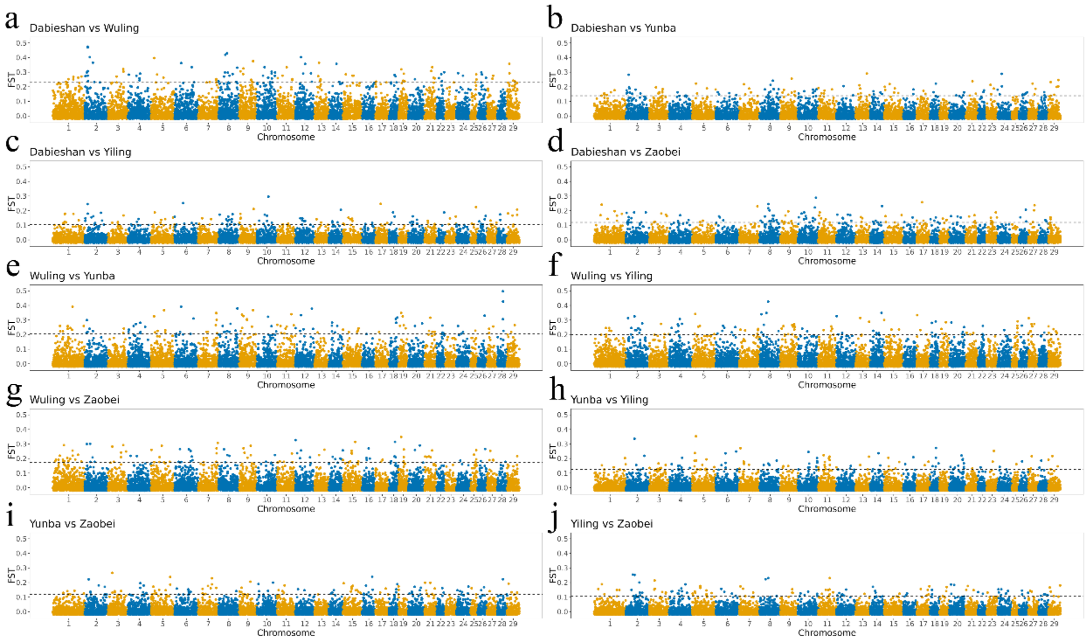

Supplementary Figure S5. Genome-wide pairwise F_{st} analysis based on large INSs and DELs among five indigenous cattle breeds: (**a**) Dabieshan vs. Wuling; (**b**) Dabieshan vs. Yunba; (**c**) Dabieshan vs. Yiling; (**d**) Dabieshan vs. Zaobei; (**e**) Wuling vs. Yunba; (**f**) Wuling vs. Yiling; (**g**) Wuling vs. Zaobei; (**h**) Yunba vs. Yiling; (**i**) Yunba vs. Zaobei; (**j**) Yiling vs. Zaobei.

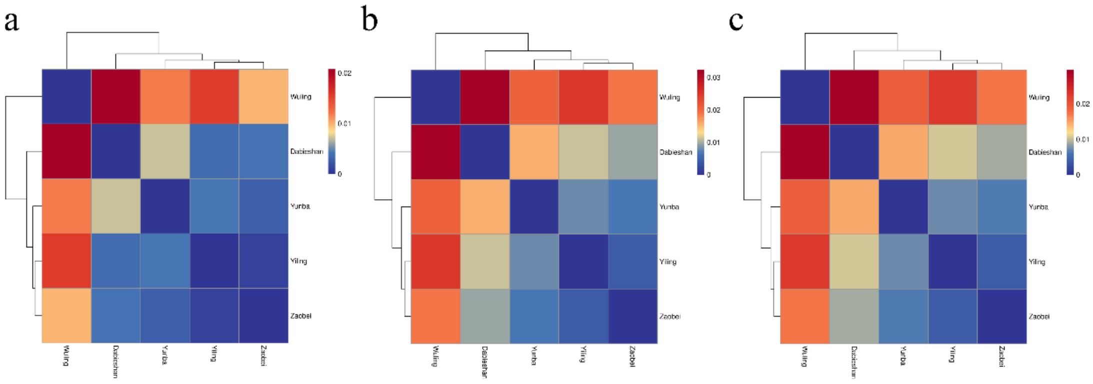

Supplementary Figure S6. Mean pairwise F_{st} values between Hubei indigenous cattle breeds based on different sizes of INSs and DELs.: (**a**) small INSs and DELs; (**b**) medium INSs and DELs; (**c**) large INSs and DELs.

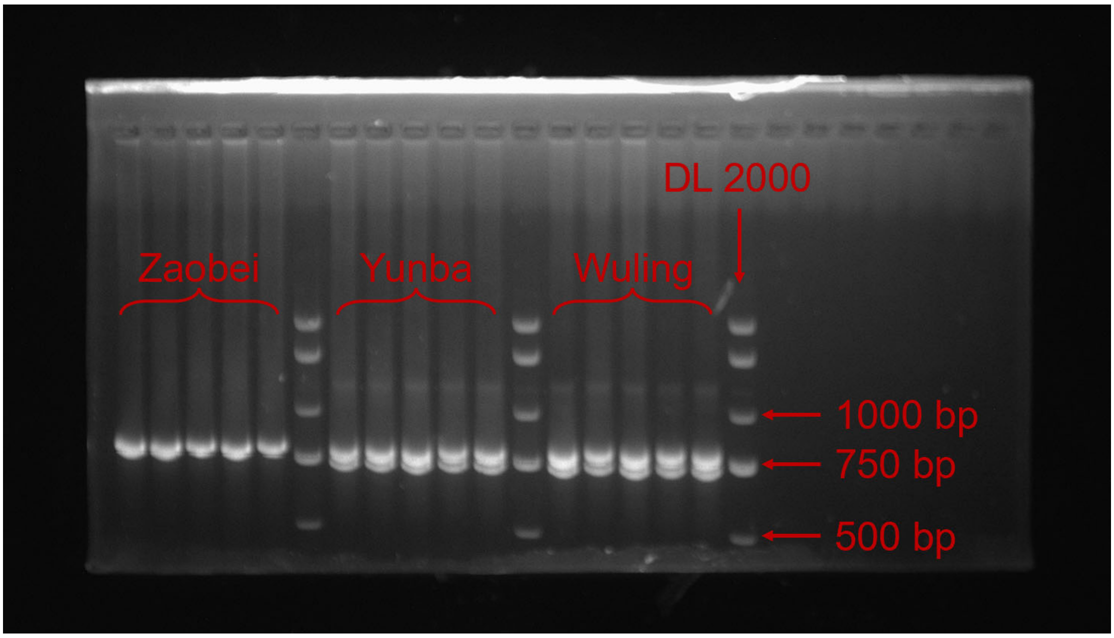

Supplementary Figure S7. PCR validation of the 67 bp insertion in the *NOTCH2* gene across different cattle breeds. Samples from left to right, separated by the DL2000 DNA marker, correspond to Zaobei, Wuling, and Yunba cattle.
