# Supplementary material for: Genomic Analysis of Indel and SV Reveals Functional and Adaptive Signatures in Hubei Indigenous Cattle Breeds
Source: Animals (Basel). 2025 Jun 13;15(12):1755. doi: 10.3390/ani15121755 (PMC12189102; doi:10.3390/ani15121755)
Supplement: Supplementary file 1 [file animals-15-01755-s001.zip › Table S1.pdf]

Table S1. Summary of GO and KEGG enrichment analyses for genes within hotspot regions.

database	gene set	description	FDR
BP	GO:0001912	positive regulation of leukocyte-mediated cytotoxicity	9.06E-06
BP	GO:0031343	positive regulation of cell killing	1.71E-05
BP	GO:0001910	regulation of leukocyte-mediated cytotoxicity	3.82E-05
BP	GO:0050911	detection of chemical stimulus involved in sensory perception of smell	4.72E-05
BP	GO:0007608	sensory perception of smell	5.61E-05
BP	GO:0031341	regulation of cell killing	6.69E-05
BP	GO:0050907	detection of chemical stimulus involved in sensory perception	7.91E-05
BP	GO:0009593	detection of chemical stimulus	1.08E-04
BP	GO:0050906	detection of stimulus involved in sensory perception	1.36E-04
BP	GO:0007606	sensory perception of chemical stimulus	1.51E-04
BP	GO:0001909	leukocyte-mediated cytotoxicity	2.09E-04
BP	GO:0002708	positive regulation of lymphocyte-mediated immunity	2.26E-04
BP	GO:0002705	positive regulation of leukocyte-mediated immunity	3.85E-04
BP	GO:0051606	detection of stimulus	3.97E-04
BP	GO:0048002	antigen processing and presentation of peptide antigen	0.001047736
BP	GO:0002706	regulation of lymphocyte-mediated immunity	0.001081901
BP	GO:0002717	positive regulation of natural killer cell-mediated immunity	0.001353702
BP	GO:0045954	positive regulation of natural killer cell-mediated cytotoxicity	0.001353702
BP	GO:0001906	cell killing	0.001843427
BP	GO:0007600	sensory perception	0.001899425
BP	GO:0002715	regulation of natural killer cell-mediated immunity	0.002551307
BP	GO:0042269	regulation of natural killer cell-mediated cytotoxicity	0.002551307
BP	GO:0002703	regulation of leukocyte-mediated immunity	0.002751861
BP	GO:0002399	MHC class II protein complex assembly	0.002872554
BP	GO:0002503	peptide antigen assembly with MHC class II protein complex	0.002872554
BP	GO:0001914	regulation of T cell-mediated cytotoxicity	0.003542593
BP	GO:0002699	positive regulation of immune effector process	0.004003507
BP	GO:0002223	stimulatory C-type lectin receptor signaling pathway	0.004003507
BP	GO:1990840	response to lectin	0.004003507
BP	GO:1990858	cellular response to lectin	0.004003507
BP	GO:0050778	positive regulation of immune response	0.004380622
BP	GO:0019882	antigen processing and presentation	0.005086031
BP	GO:0002396	MHC protein complex assembly	0.005352227
BP	GO:0002501	peptide antigen assembly with MHC protein complex	0.005352227
BP	GO:0001913	T cell-mediated cytotoxicity	0.006668137
BP	GO:0002483	antigen processing and presentation of endogenous peptide antigen	0.007189512
BP	GO:0050877	nervous system process	0.008276194

BP	GO:0002228	natural killer cell-mediated immunity	0.009719519
BP	GO:0042267	natural killer cell-mediated cytotoxicity	0.009719519
BP	GO:0002449	lymphocyte-mediated immunity	0.012188657
BP	GO:0015914	phospholipid transport	0.013932321
BP	GO:0019886	antigen processing and presentation of exogenous peptide antigen via MHC class II	0.015375396
BP	GO:0019883	antigen processing and presentation of endogenous antigen	0.01921468
BP	GO:0050776	regulation of immune response	0.022189256
BP	GO:0002478	antigen processing and presentation of exogenous peptide antigen	0.022971628
BP	GO:0007186	G protein-coupled receptor signaling pathway	0.022971628
BP	GO:0002697	regulation of immune effector process	0.030099952
BP	GO:0002495	antigen processing and presentation of peptide antigen via MHC class II	0.032123185
BP	GO:0001916	positive regulation of T cell-mediated cytotoxicity	0.032123185
BP	GO:0002504	antigen processing and presentation of peptide or polysaccharide antigen via MHC class II	0.037485094
BP	GO:0046596	regulation of viral entry into host cell	0.037485094
BP	GO:0002443	leukocyte-mediated immunity	0.039901525
BP	GO:0002709	regulation of T cell-mediated immunity	0.041394422
BP	GO:0019884	antigen processing and presentation of exogenous antigen	0.042404684
CC	GO:0009897	external side of plasma membrane	0.001843427
CC	GO:0042613	MHC class II protein complex	0.004003507
CC	GO:0042611	MHC protein complex	0.005352227
CC	GO:0009986	cell surface	0.022971628
KEGG	bta05332	graft-versus-host disease	3.27E-20
KEGG	bta04612	antigen processing and presentation	5.01E-19
KEGG	bta05330	allograft rejection	2.38E-11
KEGG	bta04940	type I diabetes mellitus	2.93E-11
KEGG	bta05320	autoimmune thyroid disease	3.75E-10
KEGG	bta05416	viral myocarditis	2.45E-09
KEGG	bta04514	cell adhesion molecules	6.19E-09
KEGG	bta04740	olfactory transduction	4.44E-07
KEGG	bta05310	asthma	4.88E-07
KEGG	bta05323	rheumatoid arthritis	9.06E-06
KEGG	bta04672	intestinal immune network for IgA production	1.02E-05
KEGG	bta05321	inflammatory bowel disease	4.72E-05
KEGG	bta04145	phagosome	4.85E-05
KEGG	bta05169	Epstein–Barr virus infection	1.36E-04
KEGG	bta05166	human T-cell leukemia virus 1 infection	1.47E-04
KEGG	bta04650	natural killer cell-mediated cytotoxicity	1.97E-04
KEGG	bta05168	herpes simplex virus 1 infection	2.91E-04
KEGG	bta04658	Th1 and Th2 cell differentiation	3.41E-04
KEGG	bta05150	staphylococcus aureus infection	4.92E-04

KEGG	bta05140	leishmaniasis	7.70E-04
KEGG	bta04659	Th17 cell differentiation	9.48E-04
KEGG	bta05322	systemic lupus erythematosus	0.002768223
KEGG	bta04640	hematopoietic cell lineage	0.004003507
KEGG	bta05145	toxoplasmosis	0.005086031
KEGG	bta01523	antifolate resistance	0.006500699
KEGG	bta02010	ABC transporters	0.023210425
FM	GO:0004984	olfactory receptor activity	4.72E-05
FM	GO:0042605	peptide antigen binding	1.10E-04
FM	GO:0038023	signaling receptor activity	6.37E-04
FM	GO:0060089	molecular transducer activity	6.37E-04
FM	GO:0004888	transmembrane signaling receptor activity	0.001081901
FM	GO:0004930	G protein-coupled receptor activity	0.001285956
FM	GO:0003823	antigen binding	0.001353702
FM	GO:0023026	MHC class II protein complex binding	0.004003507
FM	GO:0005549	odorant binding	0.004907351
FM	GO:0023023	MHC protein complex binding	0.007189512
FM	GO:0008525	phosphatidylcholine transporter activity	0.012199485
FM	GO:0031210	phosphatidylcholine binding	0.042404684
